# Encapsulation of nanoparticles into single-crystal ZnO nanorods and microrods

**DOI:** 10.3762/bjnano.5.56

**Published:** 2014-04-16

**Authors:** Jinzhang Liu, Marco Notarianni, Llew Rintoul, Nunzio Motta

**Affiliations:** 1 Institute for Future Environments and School of Chemistry, Physics, and Mechanical Engineering, Queensland University of Technology, Brisbane, 4001, QLD, Australia

**Keywords:** crystal growth, encapsulation, nanoparticles, photoluminescence, ZnO nanorods

## Abstract

One-dimensional single crystal incorporating functional nanoparticles of other materials could be an interesting platform for various applications. We studied the encapsulation of nanoparticles into single-crystal ZnO nanorods by exploiting the crystal growth of ZnO in aqueous solution. Two types of nanodiamonds with mean diameters of 10 nm and 40 nm, respectively, and polymer nanobeads with size of 200 nm have been used to study the encapsulation process. It was found that by regrowing these ZnO nanorods with nanoparticles attached to their surfaces, a full encapsulation of nanoparticles into nanorods can be achieved. We demonstrate that our low-temperature aqueous solution growth of ZnO nanorods do not affect or cause degradation of the nanoparticles of either inorganic or organic materials. This new growth method opens the way to a plethora of applications combining the properties of single crystal host and encapsulated nanoparticles. We perform micro-photoluminescence measurement on a single ZnO nanorod containing luminescent nanodiamonds and the spectrum has a different shape from that of naked nanodiamonds, revealing the cavity effect of ZnO nanorod.

## Introduction

Anisotropic growth of compound semiconductors with wurtzite crystal structure normally leads to the formation of one-dimensional (1D) structures. A typical example is ZnO that, in its wurtzite form, has the fastest growth rate over the <0001> face and has been extensively studied in terms of synthesis methods and applications. ZnO is a multifunctional material with semiconducting, photonic, and piezoelectric properties. Potential applications of ZnO 1D nanostructures include gas sensor [1], transistor [2], light-emitting device [3], optical waveguide [4], nanolaser [5], and piezoelectric power generator [6], etc. Since the first report of ZnO nanobelts in 2001 [7], methods for growing ZnO 1D nanostructures have been well developed, including high-temperature vapour-phase growth [8], low-temperature aqueous solution growth [9], and electrochemical deposition [10]. The aqueous solution growth is the least expensive one, and is scalable for production.

Single-crystal nanowires/nanorods of wide-bandgap semiconductors are ideal candidates for nanophotonic applications. The 1D geometry, dislocation-free single-crystalline nature, high index of refraction and atomically smooth surface, allow for sufficient end-facet reflectivity and photon confinement in a volume of just a few cubic wavelengths of material. Since the first report of a ZnO nanowire laser, much effort has been placed in nanophotonic research based on small-sized semiconducting nanocrystals with 1D or 2D structures. To study the photon–matter interaction within those crystals, the luminescence of the cavity material has been conventionally used as a light source. Under excitation, ZnO emits UV light at about 380 nm ascribed to its wide band gap (*E*_g_ ≈ 3.4 eV). With crystal defects such as oxygen vacancies, ZnO show visible photoluminescence under UV light excitation. Though ZnO micro/nanorods can be both light emitters and optical cavities, the drawback is that their luminescence is not tunable in terms of wavelength and efficiency. For laser applications, the visible emission of ZnO micro/nanocavities has a broad range which can be used to observe a series of optical resonances [11–12], while lasing in this range cannot be achieved. Within the narrow excitonic emission range, UV lasing from ZnO micro/nanorods has been realized [5,13], however this requires that the resonant positions sit within the narrow UV emission range. Thus the options for lasing wavelength and resonant mode orders are limited. There is a large variety of nanoparticles that have various luminescent properties and potential applications. Luminescent nanoparticles including semiconductor quantum dots, nanodiamonds (NDs) with nitrogen-vacancy (NV) centres, and dye-doped polymer nanobeads, etc., have wide applications based on their luminescent properties. Luminescent NDs can be used for magnetic sensing [14]; dye-doped polymer nanobeads can act as laser gain media [15], depending on the selection of dye molecules. Semiconductor quantum dots can be used as laser gain media as well as for quantum communications when confined within an optical cavity. The coupling of luminescent nanoparticles emission to artificial optical cavities such as 2D photonic crystals [16] and micro-spheres [17] has been studied. Single crystal nanorods as dielectric cavities have superior features in terms of their small size and excellent cavity properties. Therefore by encapsulating luminescent nanoparticles into transparent single-crystal nanorods, it is possible to develop an interesting platform for novel photonic applications.

ZnO nano/microrods are particularly interesting because they are not only optical cavities but also components for developing UV light-emitting diodes (LEDs). If luminescent quantum dots or dye-doped polymer nanobeads can be encapsulated into a ZnO nano/microrod that is integrated into a LED, the UV emission of ZnO can act as an excitation source to stimulate the visible emissions of embedded nanoparticles, providing the way to develop small-size lasers. So far, nanoparticles-filled polymers or glass fibres have been reported [18–19]. No reports exist on integrating nanoparticles into single-crystal nanorods. By sealing functional nanoparticles, such as luminescent, magnetic, and plasmonic metal materials, etc., into single crystal nanorods, we believe that many applications can be explored. Herein, we report the encapsulation of nanoparticles into ZnO nanorods by exploiting the growth habit of ZnO. A low-temperature aqueous solution growth method is used to grow ZnO nanorods. It is worth mentioning that the low-temperature growth (<100 °C) does not produce any thermal damage to the encapsulated nanoparticles, and this is important in particular for organic nanomaterials.

## Results and Discussion

### Encapsulation of nanodiamonds

**Figure 1:**
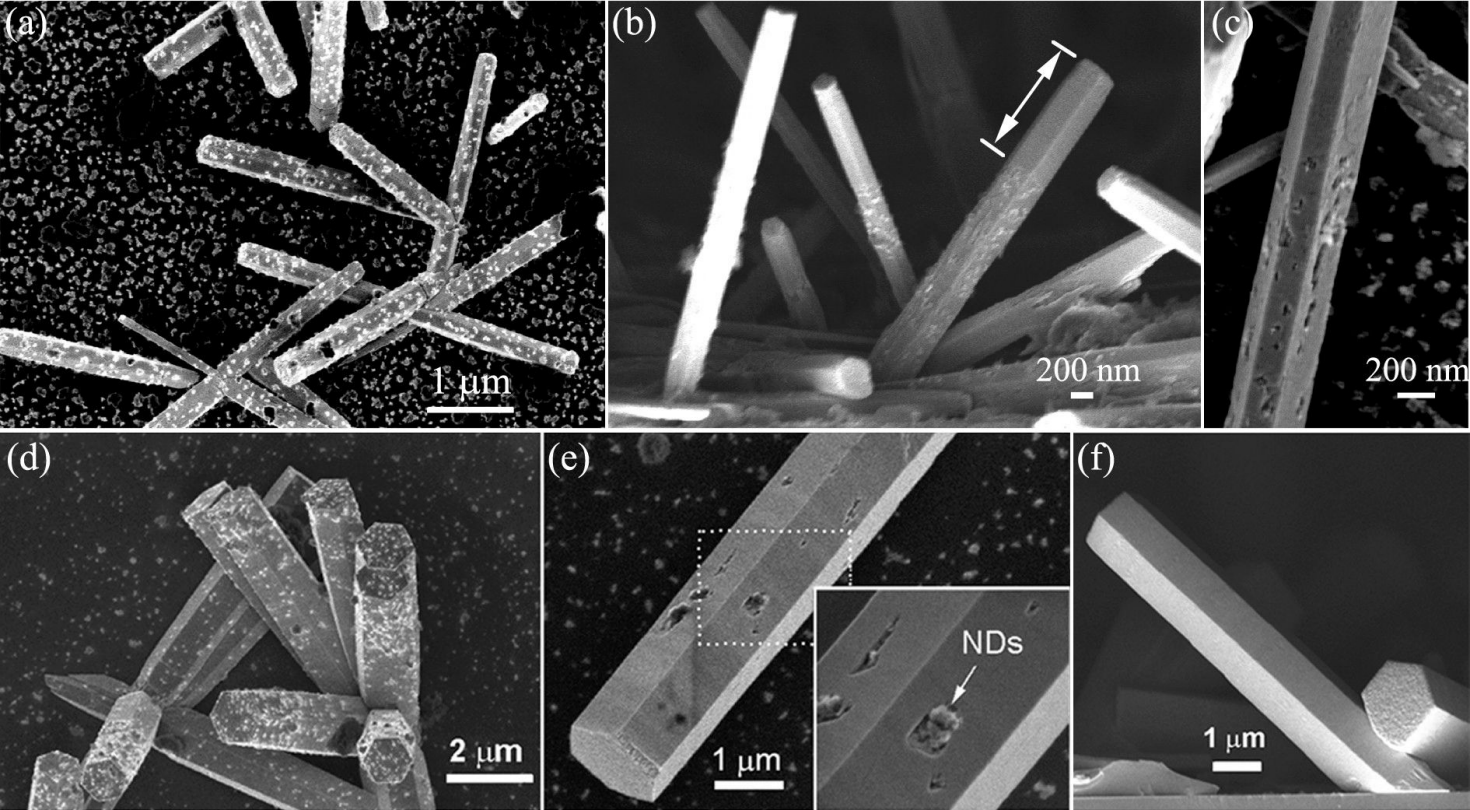
FE-SEM images showing the encapsulation of 10-nm-diameter NDs into ZnO nanorods and microrods. (a) Thin nanorods with NDs attached to their surfaces. (b) After a second growth process of ZnO nanorods. (c) A close-view of the nanorod, showing the embedment of NDs into the rod and the newly-grown section with smooth surface. (d) Relatively thick microrods with NDs in their surfaces. (e) After a second growth process, most of the NDs were encapsulated into the microrod, leaving some NDs clusters partially exposed. (f) NDs were completely encapsulated into the nanorod, given long regrowth time.

We firstly used small size NDs (10 nm size), which are non-luminescent and purchased as nanopowder, to study the encapsulation of small nanoparticles into ZnO nano/microrods, as shown in Figure 1. Two groups of samples, ZnO nanorods and microrods, used to study the embedment of NDs, are depicted in Figure 1a–c and Figure 1d–f, respectively. Figure 1a and Figure 1d show that NDs are attached to both the top and side facets of nano/microrods. The thin nanorods in Figure 1a and relatively thick microrods in Figure 1d were grown from two nutrient solutions with different chemical concentrations of 15 mM and 30 mM, respectively. The side-view image in Figure 1b shows that NDs were embedded in the nanorods after a second growth process (20 mM, 4 h). Regrowth of the nanorod along the axial direction leads to a new section with smooth surface. By measuring the length of newly-grown section, we can estimate that the second growth process results in 1.7 times increase of the length. Nevertheless, the growth over the side facets was too slow to completely bury the surface-attached NDs. The close-view image in Figure 1c shows that the incomplete encapsulation of NDs leads to holes in the nanorod surface. For ZnO microrods in Figure 1d, regrowth along the axial direction is much slower than that of the much thinner nanorods, concluded by comparing Figure 1b and Figure 1e. Note that experimental conditions for the second growth of the two samples are identical. On the other hand, for thick microrods the growth over the side facets was less suppressed as can be seen in Figure 1e, where most of the nanoparticles were completely encapsulated into the nanorods, leaving some big ND clusters partially exposed. All the surface attached NDs can be completely encapsulated into ZnO microrods if longer regrowth time (8 h) is allowed, as seen in Figure 1f. The encapsulation of 10 nm size NDs into ZnO nano/microrods indicates that luminescent quantum dots can be hosted by a ZnO nano/microrod cavity, in order to develop advanced photonic devices such as lasers and color-tunable light emitting devices. In our experiment NDs from isopropanol solution were dispersed onto both ZnO nano/microrods and a Si substrate. If a lithography technique is employed to grow ordered ZnO nanorods onto a lattice-constant-matched substrate, nanoparticles on the substrate can be avoided and the size of nanorod cavity can be well controlled in respect of light wavelength and optical cavity effect.

**Figure 2:**
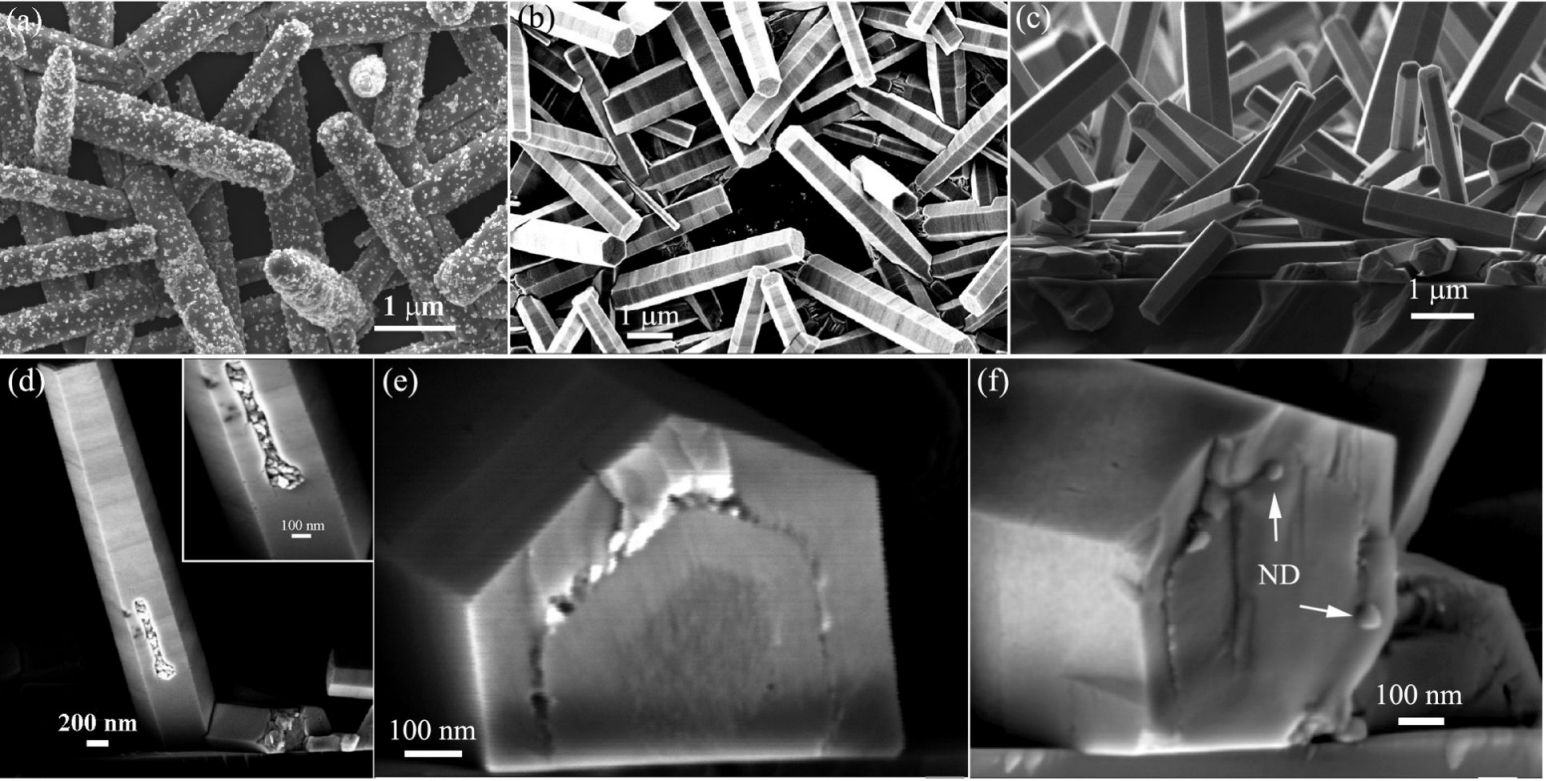
FE-SEM images showing the encapsulation of 40-nm-diameter NDs into ZnO nanorods. (a) ZnO nanorods covered with NDs. (b and c) Top- and side-view images of the nanorods after a second growth process, showing the complete encapsulation of NDs into the nanorods. (d) A nanorod shows the incomplete encapsulation of agglomerated NDs. (e and f) Cross-section images of fractured nanorods containing NDs, showing NDs inside the crystal.

Figure 2 demonstrates the encapsulation of 40 nm diameter NDs, which contain NV luminescent centres, into ZnO nanorods. We dispersed NDs from the suspension twice onto ZnO nanorods in order to increase the areal density of nanoparticles. It can be seen in Figure 2a that some NDs even agglomerate. As nanoparticles were heavily loaded onto ZnO nanorods, regrowth of ZnO can only occur over the limited bare surface. However, it is surprising to see that these surface-attached NDs were completely encapsulated into nanorods after a regrowth process (20 mM, 6 h), as evidenced by the top-view image in Figure 2b and the side-view image in Figure 1c. Among the nanorods, few show incomplete encapsulation of agglomerated NDs. as can be seen in Figure 2d. We cut the Si substrate to take side-view images. Some nanorods were fractured at the edge of the cracked substrate. The cross-sections of fractured nanorods in Figure 2e and 2f clearly show NDs completely encapsulated into the nanorods. Also, from Figure 2e we can measure that the thickness of newly-grown ZnO layer over the side facets is about 120 nm. For nanoparticles of inorganic materials, the size below 100 nm can be easily achieved. However for polymer materials it might be not easy to prepare small nanoparticles with size below 100 nm. Hence it is worth studying the encapsulation of relatively large nanoparticles into ZnO nanorods. The advantage of polymer nanobeads is that they can be multi-functional by hosting luminescent dye molecules or magnetic nanoparticles. As the growth rate over the side facets of a ZnO nanorod is much slower than that over the top facet, it would be more challenging to encapsulate large nanoparticles with size beyond the 100 nm regime into ZnO nanorod.

### Encapsulation of polymer nanobeads

**Figure 3:**
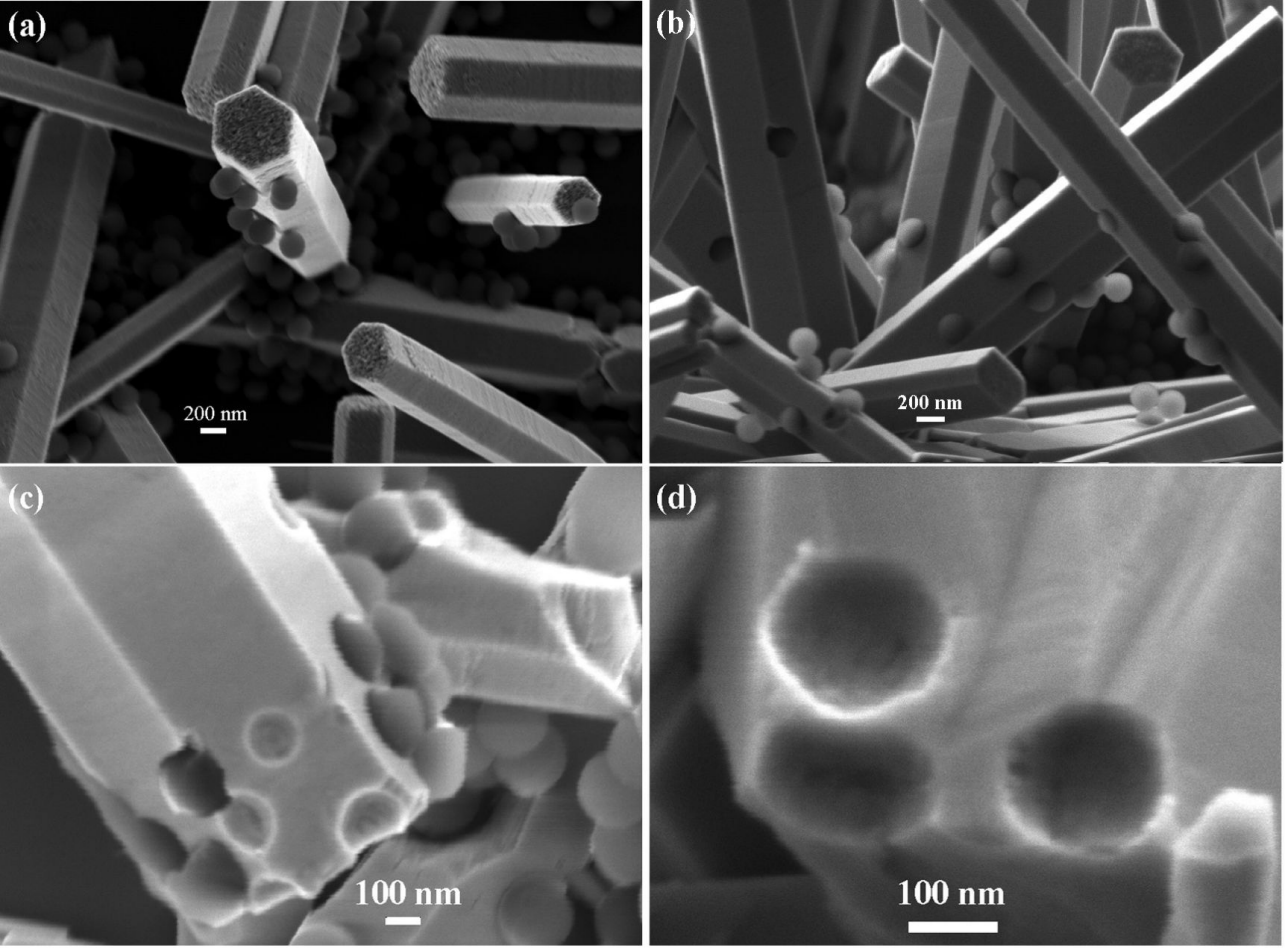
FE-SEM images showing the embedment of 200-nm-diameter polymer nanobeads in ZnO nanorods. (a) ZnO nanorods with polymer nanobeads attached to their surfaces. (b) Nanobeads were embedded in nanorods after a second growth process. These nanobeads were about half-volume exposed. (c) Intentionally fractured nanorods. (d) Close-view of the vacant sites where the nanobeads were broken off.

We used polystyrene nanobeads (diameter 200 nm) to study the encapsulation of large nanoparticles into ZnO nanorods. After dropping nanobeads aqueous suspension onto ZnO nanorods arrays and blow-drying, nanobeads were found sparsely scattered over the nanorods surfaces as seen in Figure 3a. Nanobeads attached to the top facet can be entirely encapsulated into the nanorod after a regrowth process due to the fast axial growth rate. However, those attached to the side surface were partially embedded into the nanorods after a second growth from solution (20 mM, 5 h), as seen in Figure 3b. We intentionally broke these nanorods by crushing them against a piece of bare Si for SEM imaging, as shown in Figure 3c and 3d. The close-view image in Figure 3c shows that the nanobeads were about half-volume embedded in the nanorod. Considering the nanobead radius of 100 nm, we can deduce that the thickness of newly-grown ZnO over the side facet is about 100 nm, which is consistent with the thickness of newly-grown layer revealed by Figure 2c. Some nanobeads embedded into the nanorod were detached when the nanorods were mechanically fractured. This exposes the bowl-shaped pits where the nanobeads once were. A close-view image of these voids in Figure 3d shows that their inner surface is rather smooth, which means the embedded nanobeads are tightly surrounded by ZnO. We believe that no chemical bonds were formed at the interface between nanobeads and ZnO, and the interaction is van der Waals force.

**Figure 4:**
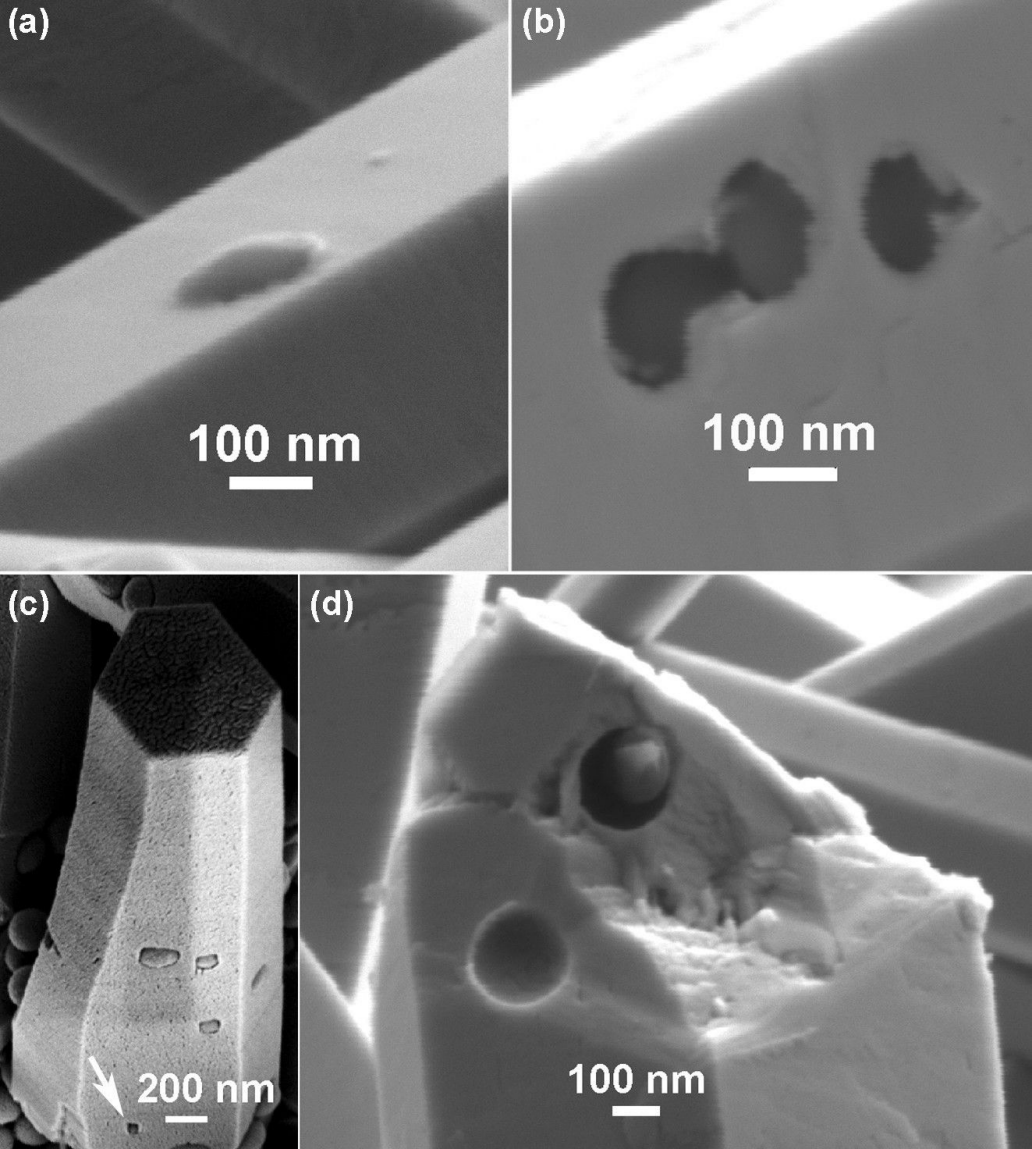
FE-SEM Images showing the encapsulation of nanobeads into ZnO nano/microrods after different regrowth processes. (a) Most part of the nanobead is embedded into a nanorod. (b) Nanobeads are deeper embedded into a nanorods, leaving holes in the surface. (c) A thick microrod showing that the embedded nanobeads are slightly exposed. (d) A fractured microrod showing a completely encapsulated nanobead inside.

The polymer nanobeads can be embedded deeper or even completely incorporated into ZnO crystal, depending on how much the nanorod is regrown. Figure 4a shows the nanorod after a regrowth process of 8 h. Such a long regrowth process leads to an overgrowth thickness more than 120 nm but less than 200 nm, leading to a deep embedding of nanobeads into the ZnO nanorod. In Figure 4b, the nanobeads were embedded even deeper after a 12 h regrowth (thickness above 200 nm) of the nanorod. The deeply embedded nanobeads leave holes in the nanorod surface of about 100 nm across, much smaller than the nanobeads (200 nm). It is conceivable that the holes will gradually fill in and finally close if the nanorod continues to grow. Figure 4c shows a nanorod in which some nanobeads are slightly exposed and one is almost encapsulated into the nanorod bulk as indicated by an arrow. In the aqueous solution most of the Zn^2+^ ions are condensed into ZnO within 4 h, and the chemicals are exhausted if the growth duration exceeds 12 h. Hence the 200 nm diameter nanobeads cannot be completely sealed into the nanorods by simply prolonging the growth time. To further thicken the nanorods, we used a three-step growth process. The nanorods after the first growth step were used to support nanobeads. After the second growth for 4 h, which is insufficient to completely encapsulate the nanobeads into ZnO nanorods, the sample was transferred to a fresh nutrient solution for the third-step growth for 4h. The fractured nanorod in Figure 4d reveals a nanobead entirely encapsulated into the nanorod.

### Growth mechanism

The growth of ZnO in aqueous solution can be described as an epitaxial growth process. The precursors Zn(NO_3_)_2_ and HMTA in water lead to chemical reactions are as follows [20]:

[1]
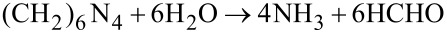


[2]
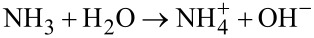


[3]
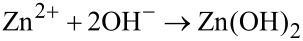


[4]
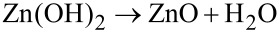


**Figure 5:**
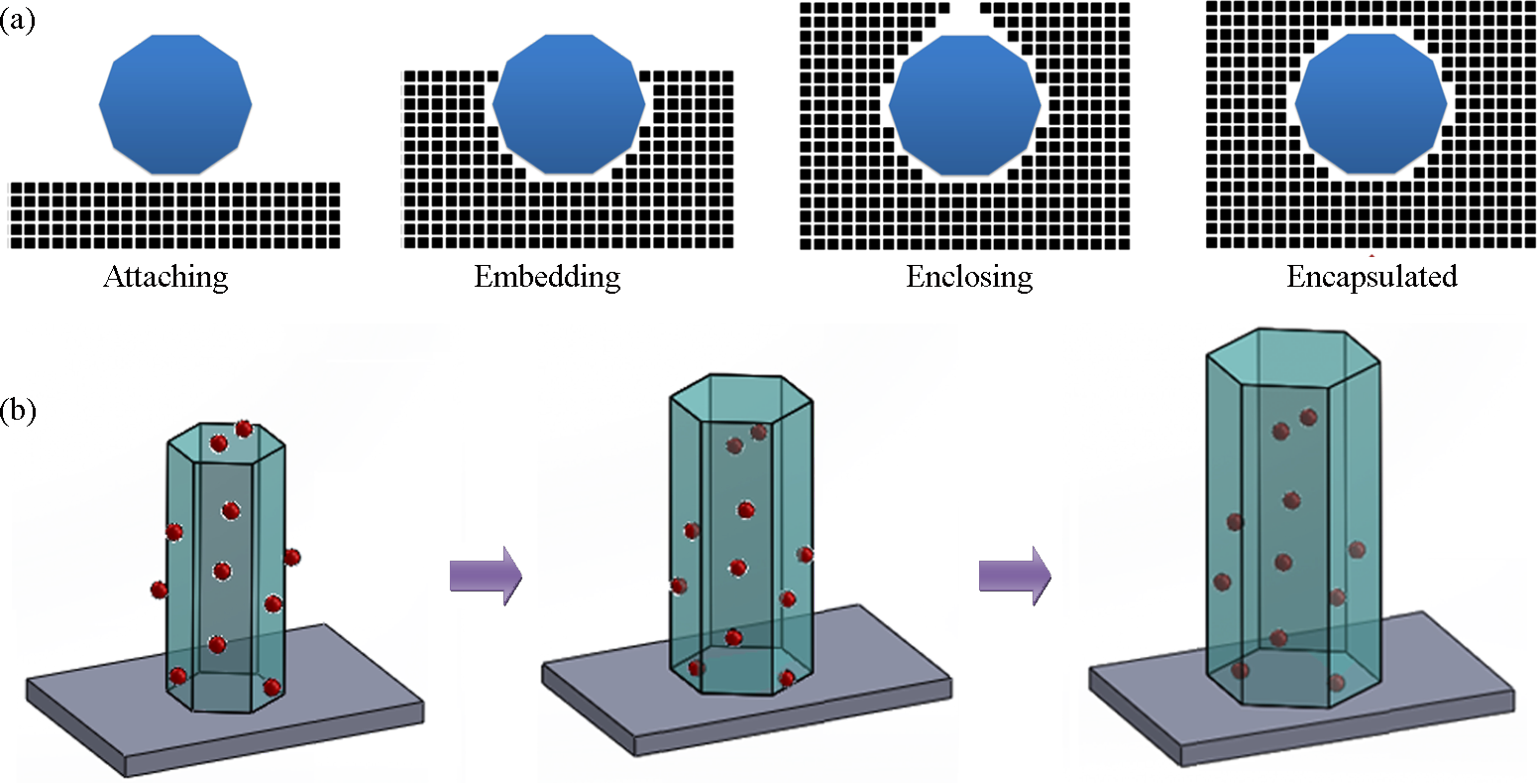
(a) Illustration of the encapsulation process of a surface-attached nanoparticle into the growing crystal. (b) Encapsulation of nanoparticles into a ZnO nanorod by regrowing the nanorod.

Hence, Zn^2+^ and OH^−^ are the growth species contributing to the growth of ZnO. When the ZnO nanorod is in the nutrient solution, the growth species deposited onto the surface contribute to the stack of atomic layers, making the nanorod grow thicker and longer while maintaining the single-crystal feature and hexagonal shape (Figure S1, Supporting Information File 1). This growth process can be described as solution homoepitaxy [21–22]. By crushing ZnO nano/microrods containing NDs onto a bare Si, we were able to expose NDs in the fracture section. The smooth and continuous surface around those exposed NDs or tiny cavities with NDs off further reveals the single-crystal character of the rod after two-step growth process (Figure S2, Supporting Information File 1). Figure 5a illustrates the encapsulation of a nanoparticle into a growing crystal. Initially the nanoparticle attaches to the crystal surface. With atomic layers stacking and spreading over the surface, the nanoparticle is embedded in newly-grown crystal. Step by step, the crystal grows around the nanoparticle enclosing it into a perfectly matching cavity. When the overgrowth is nearly completed a hole above the nanoparticle could still be present. The epitaxial growth continues and the hole gradually shrinks and finally closes, sealing the nanoparticle in crystal. Figure 5b is a 3D view of the encapsulation process. The top (0001) facet of ZnO nanorod is known to have the fastest growth rate, hence nanoparticles at the top face can be quickly encapsulated. Complete encapsulation of nanoparticles attached to the side facets into the nanorod can be achieved if the newly-grown layer thickness is beyond the nanoparticle size.

### Micro-photoluminescence of a single ZnO nanorod containing luminescent nanodiamonds

**Figure 6:**
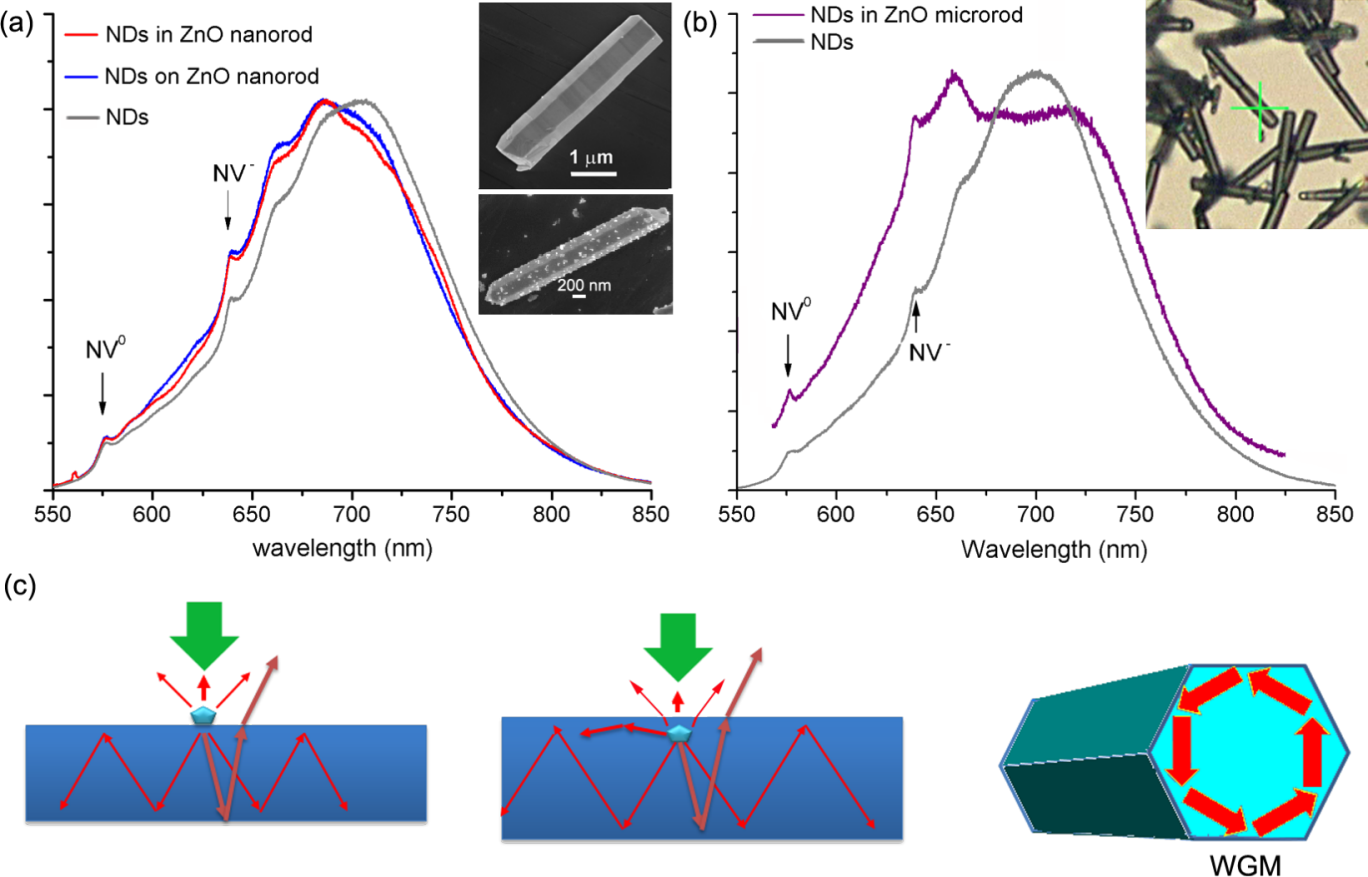
(a) Micro-PL spectra taken from a single ZnO nanorod containing NDs, a nanorod with NDs in the surface, and cluster of naked NDs, respectively. The insets show typical SEM images of two fractured ZnO nanorods lying on bare Si. One nanorod contains NDs and the other one has NDs attached to its surface. (b) Micro-PL spectra of a single ZnO microrod containing NDs and naked NDs. The inset shows an optical photograph taken during the PL measurement. The relatively thick rod marked by a cross was targeted for PL measurement. (c) Illustrations for light propagations when the emission from a ND interacts with the rod cavity.

The 40 nm size NDs with NV luminescent centers exhibit red photoluminescence (PL) when excited by a green laser. The results of micro-PL measurements on a single ZnO nanorod containing luminescent NDs, a ZnO nanorod with NDs attached to its surface, and naked NDs dispersed from solution on a steel substrate are shown in Figure 6a. The insets show two fractured ZnO nanorods lying on Si substrates. One nanorod has NDs embedded inside and the other one has naked NDs attached to its surface. The three PL spectra, with peak maximum normalized, have two small characteristic peaks of the NV defect at 576 and 638 nm, corresponding to charge states of NV^0^ and NV^−^, respectively. The PL spectrum of naked NDs shows peak position of emission band at 705 nm. However, for those NDs in contact with a ZnO nanorod, either embedded or in the surface, their PL emission peak is blue-shifted to 686 nm. The laser energy is insufficient to produce PL of ZnO, ruling out the influence of ZnO luminescence in causing the shift of NDs emission. In Figure 6b, the PL spectrum of NDs in a thick ZnO microrod shows a different shape compared to that of naked NDs. The inset in Figure 6b is an optical microscope image, in which the relatively thick rod marked by a cross was the target for collecting PL spectrum. The diameter of this rod is estimated to be 1.5–2.0 μm, whereas those in Figure 6a are 0.6–1.0 μm. In micro-PL measurement, the laser beam with a spot size of about 1 μm was perpendicular to the nanorod and NDs inside or attached to the nanorod were excited to produce red luminescence. ZnO nanorod is transparent to visible light and emissions from NDs can transmit into ZnO nano/microrod to undergo multiple reflections. ZnO is a birefringent crystal with a refractive index of about 2.0 in the visible range, indicating the total refraction angle of light ray in ZnO is about 30 °C. Due to the waveguide effect partial photons would propagate along the nanorod axis direction. Hence inside the nanorod light rays refracted to top upper facet have contribution to the PL signal (Figure 6c). Light rays with different polarizations have different transmittance when refracted from ZnO to air. The blue-shift of emission band of NDs inside or in the surface of a ZnO nanorod could be attributed to the optical cavity effect of ZnO nanorod. One possible light propagation path is a close loop in the rod cross-section, as show in the right hand in Figure 6c. That means, the nanorod is treated as a 2D cavity and the light ray strikes at the center of boundary at 60° incident angle to circulate in a hexagon loop, leading to whispering-gallery mode (WGM) resonances when the length of loop is an integer multiple of light wavelength. Normally, the larger the cavity size, the higher the resonance quality [23–24]. In the PL spectrum, the resonant peaks would broaden and peaks will be more separated with reducing the nanorod diameter. In Figure 6b, the PL spectrum of a relatively thick ZnO microrod containing NDs shows not only the blue-shift of the luminescence band but also the suppressed intensity of the band central region. The shoulder peak at 660 nm is pronounced, which can be explained by the resonant cavity-induced enhancement. The thickness of this microrod is estimated to be 1.5–2.0 μm, which is more suitable to produce discernible WGMs compared to those thinner nanorods with diameters smaller than 1 μm. Light emission from NDs is coupled to the microrod cavity and WGM resonances behind the PL spectrum leads to enhancement of luminescence at positions aside the emission band peak, making the PL peak shift and the band shoulders prominent. Fabry–Pérot type resonances originated from photons bouncing between two opposite side facets of the nano/microrod cannot be observed due to the short inter-distance. If vertical ZnO nano/microrods containing luminescent nanoparticles are illuminated by an excitation laser at the top, we believe that much stronger PL signal can be collected due to the waveguide effect and Fabry–Pérot type resonances or even lasing can be achieved.

## Conclusion

In summary, we have demonstrated the encapsulation of nanoparticles into single crystal ZnO nanorods by exploiting crystal growth. Nanodiamonds and polymer nanobeads are used as examples to study this process for nanoparticles with different sizes and shapes into ZnO nanorods grown in aqueous solution at low temperature. Our two-step aqueous epitaxial growth process results in the full encapsulation of 10 nm and 40 nm nanodiamonds. The same two-step process results only in a partial embedment of 200 nm diameter polymer nanobeads. Complete encapsulation of these relatively larger nanobeads into ZnO nanorods can be achieved by a further epitaxial growth of ZnO via a three-step process using the same solution method. Our study indicates that in principle any kinds of nanoparticles, both inorganic and organic, can be incorporated into ZnO nanorods. The epitaxial growth over ZnO surface leads to the encapsulation of nanoparticles into the crystal while maintaining the single crystal feature. We believe that nanoparticles can be encapsulated into not only ZnO, but also other functional crystals grown by the epitaxy process. Micro-PL measurements on a single ZnO nanorod containing luminescent NDs demonstrates that the light emission from NDs can be coupled to the nanorod cavity, resulting in shift of the PL emission peak and change of the PL spectrum shape.

This low temperature process opens the way to the encapsulation of nanoparticles made of polymers and even biomaterials, which would degrade at high temperature, creating a new platform for nano-devices, nano-detectors, and applications in nano-medicine.

## Experimental

ZnO nanorods were grown at 90 °C onto Si substrates in an aqueous solution containing zinc nitrate and hexamethylenetetramine (HMTA) with molar ratio of 1:1. A piece of bare Si was put face-down floating on top of the nutrient solution surface. The strategy to encapsulate nanoparticles into ZnO nanorods is through a multi-step growth process. First, ZnO nanorods are grown onto the substrate; second, nanoparticles are attached to the surface of nanorods; Third, the nanorods are grown again in the solution to incorporate the nanoparticles. The growth duration was set 4 h for the first-step growth, but different concentrations of zinc nitrate and HMTA in the solution were used to control the nanorod thickness. To grow thin nanorods, 15 mM Zn^2+^ in the solution was used. Thick nanorods were grown in the 30 mM solution. After growth, the sample was rinsed with DI water and dried. Afterwards, nanoparticle suspension was dropped onto the substrate covered with nanorods, and then rinsed. This leads to the attachment of nanoparticles onto the nanorods surfaces by van der Waals force. The sample was dried at 50 °C, followed by a second growth of ZnO nanorods in the nutrient solution containing 20 mM Zn^2+^. The regrowth duration was 4–12 h, and different nanoparticles were used: two types of diamond nanoparticles with average diameters of 10 nm and 40 nm, respectively, and polymer nanobeads with mean size of 200 nm. Nanopowder of NDs with mean size of 10 nm (Aldrich Sigma) was dispersed into isopropanol (0.2 mg/mL) to prepare a suspension. An aqueous suspension of 40 nm diameter NDs (0.1 mg/mL) with NV luminescent centres was purchased from Adamas Nanotech. Polystyrene nanobeads (200 nm size) dispersed in water (0.5 mg/mL) were purchased from Polyscience Inc. Field-emission scanning electron microscopy (FE-SEM, Zeiss Sigma) was employed to study the morphologies of ZnO nanorods and embedded nanoparticles. For micro-PL measurement, ZnO nanorod arrays were crushed against a bare Si, to produce fractured nanorods lying on the surface. A micro-Raman/PL spectrometer (Renishaw inVia) with 532 nm laser was employed to study the PL of single ZnO nanorod containing NDs.

## Supporting Information

File 1 Additional figures.
