# Supplementary material for: Encapsulation of nanoparticles into single-crystal ZnO nanorods and microrods
Source: Beilstein J Nanotechnol. 2014 Apr 16;5:485–93. doi: 10.3762/bjnano.5.56 (PMC3999869; doi:10.3762/bjnano.5.56)
Supplement: File 1 — Additional figures. [file Beilstein_J_Nanotechnol-05-485-s001.pdf]

Supporting Information

for

Encapsulation of nanoparticles into single-crystal ZnO nanorods and microrods

Jinzhang Liu*, Marco Notarianni, Llew Rintoul and Nunzio Motta

Address: Institute for Future Environments and School of Chemistry, Physics, and Mechanical Engineering, Queensland University of Technology, Brisbane, 4001, QLD, Australia

Email: Jinzhang Liu* - jinzhang.liu@qut.edu.au

*Corresponding author

Additional figures

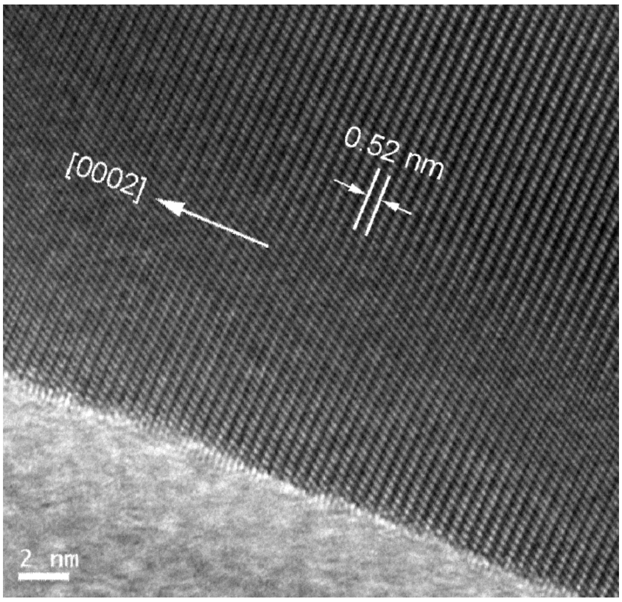

Figure S1: High resolution transmission electron microscopy image of a ZnO nanorod grown by the aqueous solution method, showing the single crystal feature and growth direction along [0001].

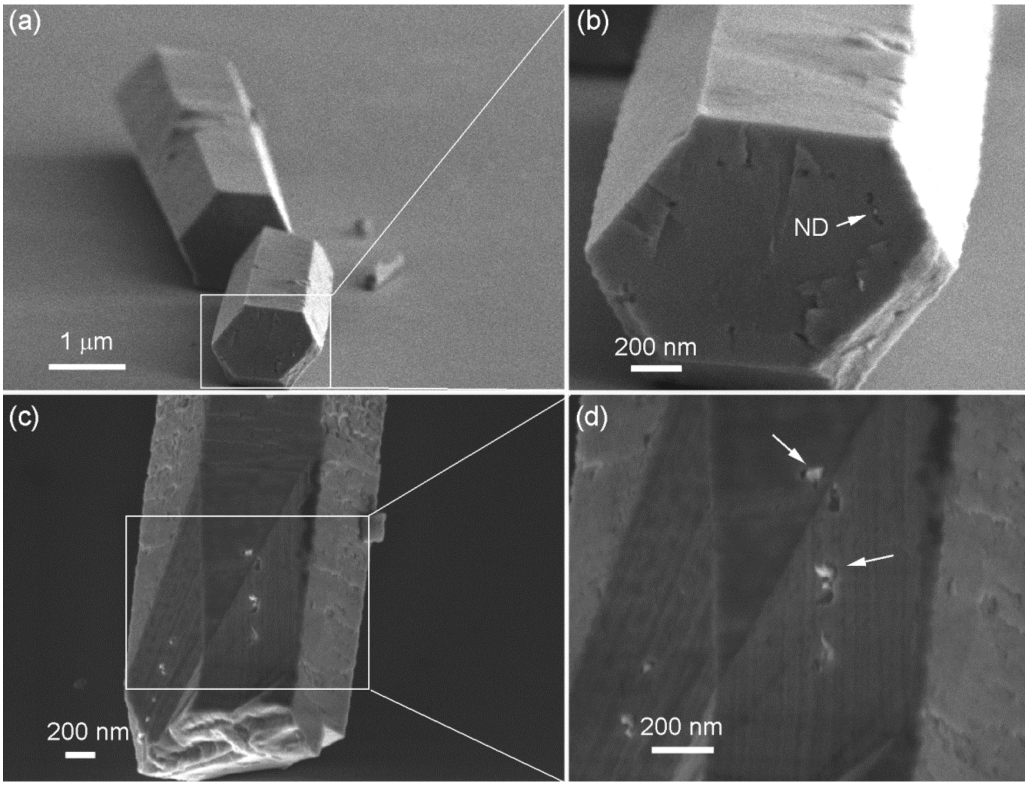

Figure S2: FE-SEM images of fractured ZnO microrods lying on Si substrate. (a) Tilted-view image. (b) A close view image of the cross-section, revealing tiny cavities and an exposed ND. (c) Top-view image of a ZnO microrod with NDs in the fractured section. (d) A close view for exposed NDs.
